# Supplementary figures and images for: Aicardi-Goutières syndrome-associated gene SAMHD1 preserves genome integrity by preventing R-loop formation at transcription–replication conflict regions
Source: PLoS Genet. 2021 Apr 15;17(4):e1009523. doi: 10.1371/journal.pgen.1009523 (PMC8078737; doi:10.1371/journal.pgen.1009523)

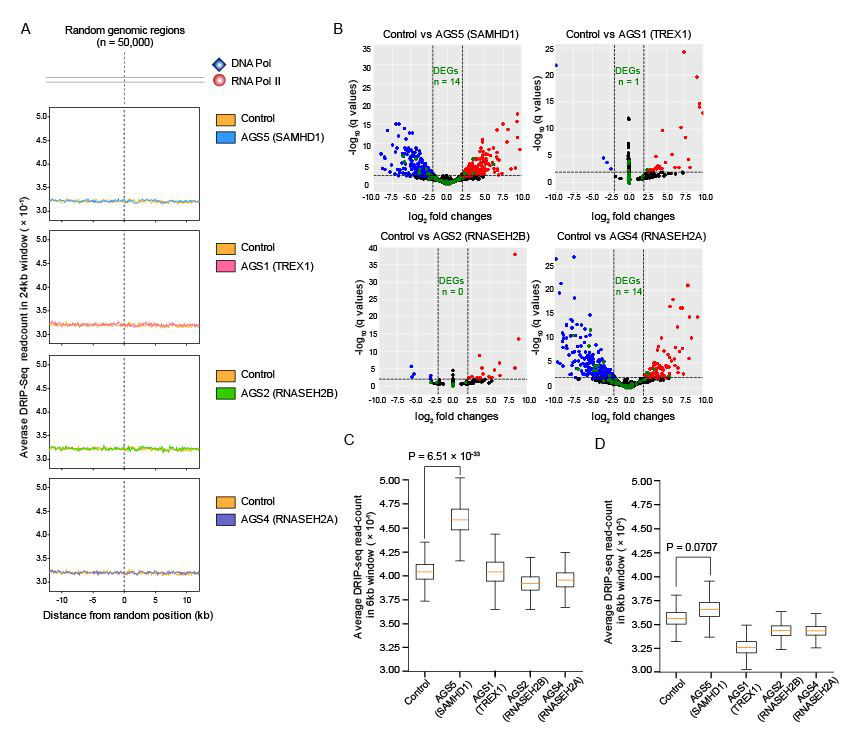

Supplement: S1 Fig — A, Average mapped DRIP-seq read accumulation around random genomic position (n = 50,000) in fibroblasts from control (yellow) and AGS patients with the indicated gene mutations (blue, SAMHD1; pink, TREX1; green, RNASEH2B; purple, RNASEH2A). For individual graph, the line indicates the mean DRIP-seq read-count of each genomic region of 24 kb windows centered on random genomic position. B, Volcano plot of RNA-seq transcriptome data displaying the pattern of gene expression values for fibroblasts from control and AGS patients (AGS5, AGS1, AGS2, and AGS4). Significantly differentially expressed genes (DEGs) (|log2(fold change)| >2 and adjusted P value <0.01) are highlighted in red (upregulated) or blue (downregulated). None of the DEGs are highlighted in black. Selected genes used for DRIP-seq analysis (n = 467) are highlighted in dark green. The number of DEGs among selected genes used for DRIP-seq analysis for each AGS patient are indicated. C, Box graph indicating quantified average DRIP-seq read-count across the 6 kb window upstream from replication origins of the gene bodies (n = 727), where HO collision sub-regions exist. D, Box graph indicating quantified average DRIP-seq read-count across a 6-kb window downstream from replication origins of the gene bodies (n = 727), where CD collision sub-regions exist. In C and D, the orange line at the center of the boxes indicates the median, and the boxes indicate 1st and 3rd quartiles. Statistical significance was assessed using Fisher’s exact test followed by Bonferroni correction. (TIF) [file pgen.1009523.s001.tif]

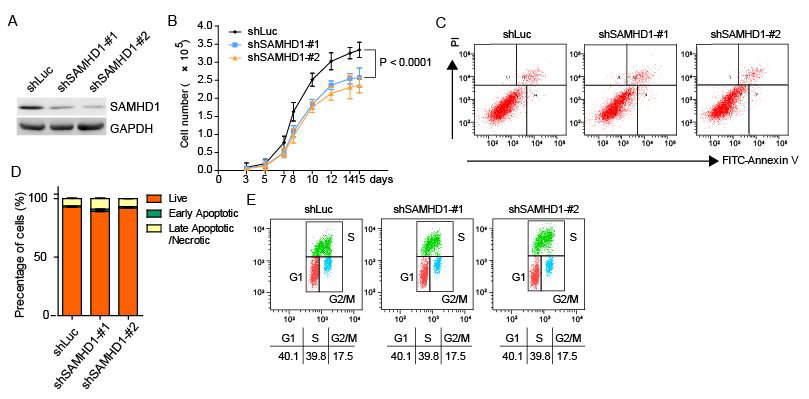

Supplement: S2 Fig — A, SAMHD1 in U2OS cells transfected with vectors expressing shRNA targeting luciferase control (shLuc) and two different SAMHD1 UTRs (shSAMHD1-#1 and shSAMHD1-#2). B, In vitro cell proliferation assay of SAMHD1-depleted or control U2OS cells. Data represent the mean ± SD, n = 3. The P value was calculated according to two-way ANOVA. C, Representative flow plot of Annexin V-FITC assay of SAMHD1-depleted and control U2OS cells. Apoptosis was determined by staining cells with FITC-conjugated Annexin V and PI, followed by flow cytometry analysis. Four populations are indicated as Q1, necrotic; Q2, late apoptosis; Q3, live; and Q4, early apoptotic. D, Quantification of Annexin V-FITC apoptosis assay results showing the percentage of cell death modes: live cells, early apoptosis, and late apoptosis/necrosis in SAMHD1-depleted and control U2OS cells. Statistical significance was assessed by two-way ANOVA. Data represent the mean ± SEM (n = 3, P < 0.001). E, Representative cell cycle profile analysis by flow cytometry following labeling of cells with BrdU. SAMHD1-depleted and control U2OS cells were pulsed with BrdU. The BrdU-positive population was followed over time as it transitioned through the cell cycle. (TIF) [file pgen.1009523.s002.tif]

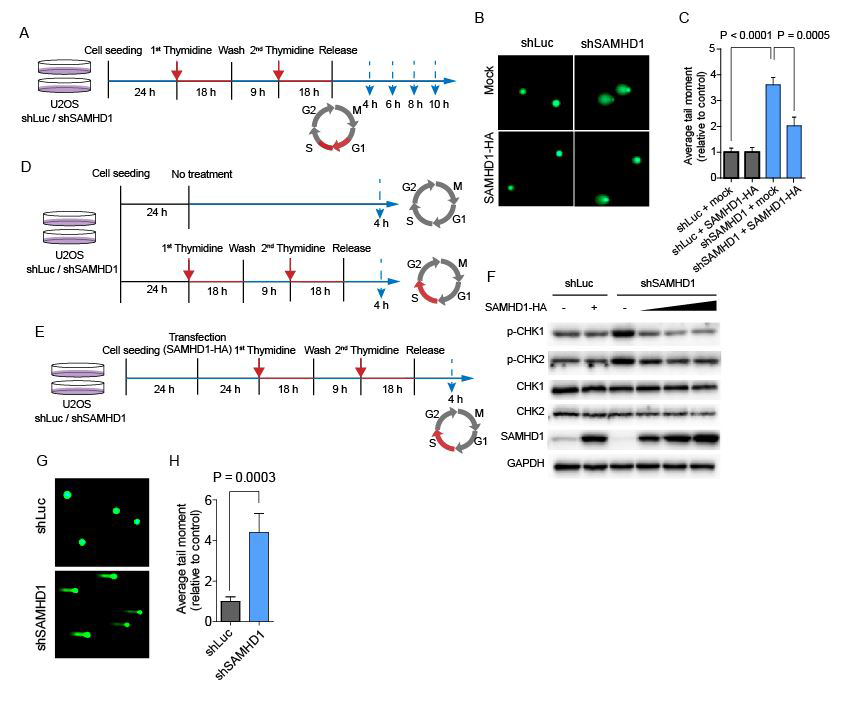

Supplement: S3 Fig — A, Summary of experimental design for Fig 2C, representing experimental process of double thymidine block followed by time course sample harvest. B, Representative images of comet assays performed under alkaline electrophoresis conditions in SAMHD1-depleted and control U2OS cells, in the absence or presence of ectopic SAMHD1-HA expression. C, Quantitative analysis of comet tail moment lengths for each condition described in B. Statistical significance was assessed using two-tailed Student’s t-test (n >50). D, Summary of experimental design for Fig 2F, representing experimental process to obtain heterogeneous (Asyn) or S phase-synchronized cellular population by double thymidine block. E, Summary of experimental design for F, representing experimental process of SAMHD1-HA reconstitution followed by double thymidine block to obtain S phase-synchronized cellular population. F, Immunoblots of CHK1 phosphorylated on S345 (p-CHK1), CHK2 phosphorylated on T68 (p-CHK2), CHk1 and CHk2 in S phase-synchronized SAMHD1-depleted and control U2OS cells, in absence or presence of ectopic SAMHD1-HA expression in a does dependent manner (n = 3). G, Representative images of comet assays performed under neutral conditions in SAMHD1-depleted and control U2OS cells. H, Quantitative analysis of comet tail moment lengths for each condition described in G. Statistical significance was assessed using two-tailed Student’s t-test (n >50). (TIF) [file pgen.1009523.s003.tif]

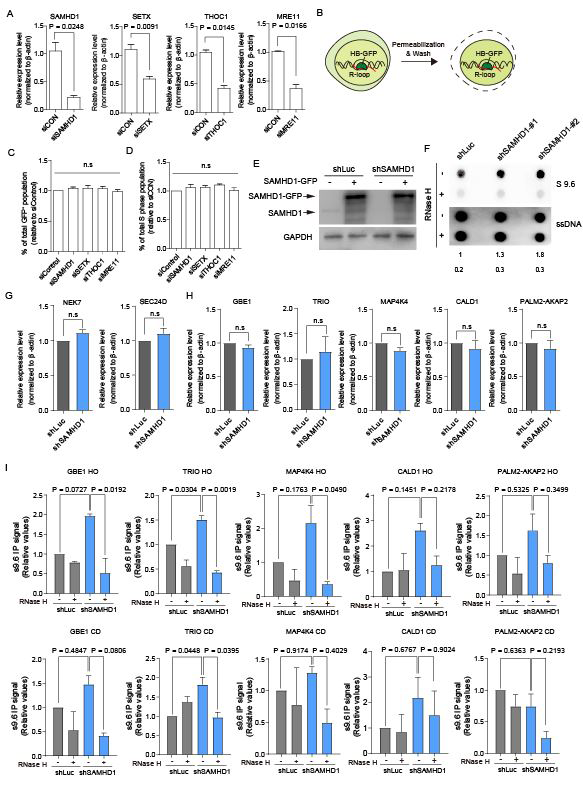

Supplement: S4 Fig — A, Indicated gene expression as measured by RT-qPCR in control (siControl) or indicated siRNAs treated U2OS cells. Data represent mean ± SEM, n = 3, P values were calculated according to two-tailed Student’s t-test. B, Schematic representation of HB-GFP retention assay. C, Quantification analysis of HB-GFP retention assay co-stained with PI. Data represent mean ± SEM percentages of GFP positive cell population. Statistical significance was assessed using two-tailed Student’s t-test (n = 3). P > 0.05; n.s, not significant. D, Quantification analysis of HB-GFP retention assay co-stained with PI. Data represent mean ± SEM percentages of S phase cell population. Statistical significance was assessed using two-tailed Student’s t-test (n = 3). P > 0.05; n.s, not significant. E, Immunoblots of SAMHD1 in cells described in Fig 3G, showing ectopic expression of GFP-tagged SAMHD1 in SAMHD1-depleted and control U2OS cells. F, Dot blot analysis of R-loops in genomic DNA extracted from SAMHD1-depleted and control U2OS cells (n = 3). The total cellular genomic DNA from each cell line was incubated with or without RNase H in prior to loading for blotting, as indicated. The genomic DNA loaded membrane were processed to immunoblotting with anti-S9.6 and anti-ssDNA antibodies to verify equivalent DNA loading. Fold-induction was normalized to the control U2OS cells by quantifying the dot intensity of the blots and calculating the ratios of S9.6 signal to total amount of genomic DNA.G and H, Indicated gene expression as measured by RT-qPCR in control or SAMHD1-depleted U2OS cells. Data represent mean ± SEM, n = 3, P values were calculated according to two-tailed Student’s t-test. P > 0.05; n.s, not significant. I, DRIP-qPCR using the anti S9.6 antibody at GBE1 HO, GBE1 CD, MAP4K4 HO, MAP4K4 CD, CALD1 HO, CALD1 CD, PALM2-AKAP2 HO and PALM2-AKAP2 CD are shown in SAMHD1-depleted and control U2OS cells. Pre-immunoprecipitated samples were untreated (-) or treated (+) with RNase H, as indic [file pgen.1009523.s004.tif]

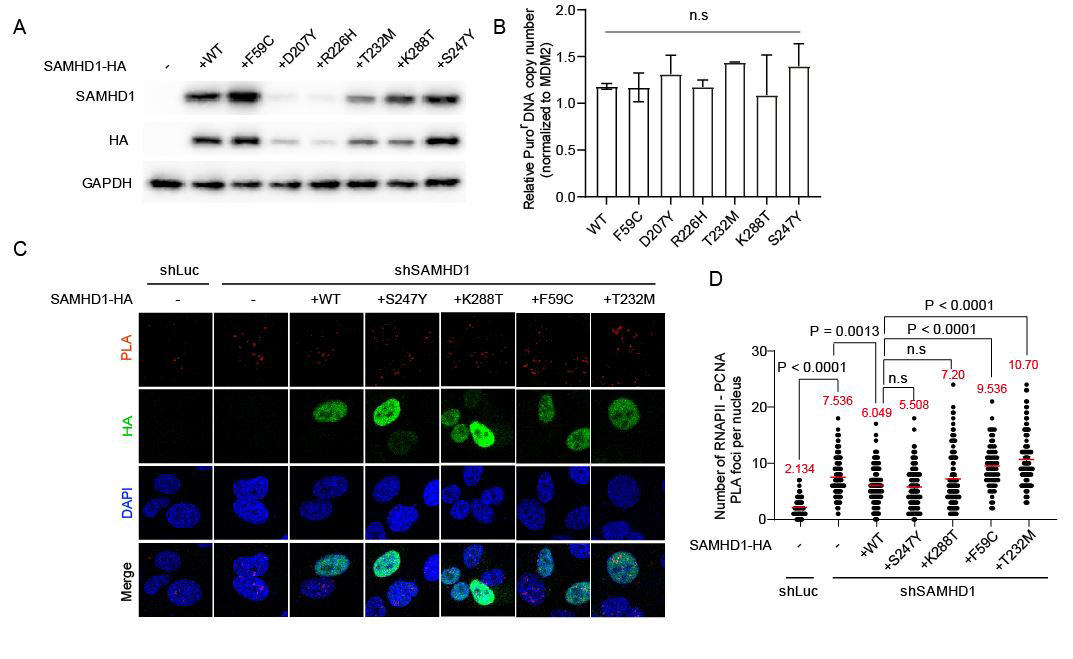

Supplement: S5 Fig — A, Immunoblots of SAMHD1 in U2OS cells transfected with vector expressing indicated SAMHD1 mutants tagged with HA. B, Genomic DNA extracted from U2OS cells described in A were analyzed by quantitative PCR. The relative DNA copy number for each vector was determined by Puromycin resistance sequence and normalized to MDM2. Data represent mean ± SEM, n = 2, P values were calculated according to two-tailed Student’s t-test. P > 0.05; n.s, not significant. C, Representative images of proximity ligation assay (PLA) between PCNA and RNA polymerase II (RNAPII) in S phase synchronized SAMHD1-depleted and control U2OS cells, in the absence or presence of indicated SAMHD1 mutant ectopic expression (SAMHD1-HA) (n = 2). Cells were subjected to PLA (red), and co-stained with HA (green) and DAPI (blue). D, Quantification analysis of number of PLA foci per nucleus in C. The mean value for each data is shown as a red line. Statistical significance was assessed using the two-tailed Mann-Whitney U test (n > 70). (TIF) [file pgen.1009523.s005.tif]

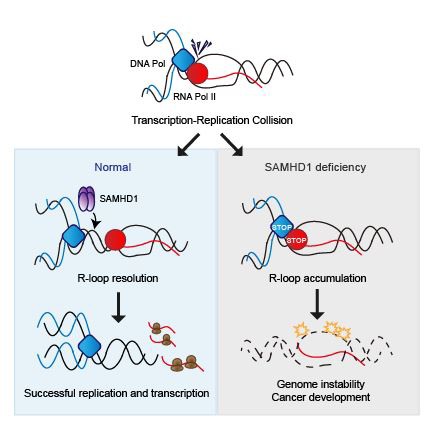

Supplement: S6 Fig — In normal cells (left), R-loops formed by transcription-replication collision, are resolved by SAMHD1, which allows successful DNA replication and RNA transcription. In SAMHD1-deficient cells (right), unresolved R-loops at transcription-replication conflict regions showed genomic instability. (TIF) [file pgen.1009523.s006.tif]
