# Supplementary material for: Aicardi-Goutières syndrome-associated gene SAMHD1 preserves genome integrity by preventing R-loop formation at transcription–replication conflict regions
Source: PLoS Genet. 2021 Apr 15;17(4):e1009523. doi: 10.1371/journal.pgen.1009523 (PMC8078737; doi:10.1371/journal.pgen.1009523)
Supplement: S1 Table — (DOCX) [file pgen.1009523.s007.docx]

**Park and Ryoo et al.**

**Supplemental Tables**

**S1 Table.**  **Oligonucleotides used in this study**.

| **Primers used for qPCR** | **Sequence 5’ to 3’** |
| --- | --- |
| *THOC1 Fwd* | CAGAGACAAGGGAACACATG |
| *THOC1 Rev* | CAGAAGGAGGCGGTAATTCC |
| *SETX Fwd* | CTTCATCCTCGGACATTTGAG |
| *SETX Rev* | TTAATAATGGCACCACGCTTC |
| *SAMHD1 Fwd* | CTGGTGCGAGCACTTGCCGA |
| *SAMHD1 Rev* | TGGGCGAGCCCGTGGGATAA |
| *MRE11 Fwd* | GCCTTCCCGAAATGTCACTA |
| *MRE11 Rev* | TTCAAAATCAACCCCTTTCG |
| *β-actin Fwd* | AGAGCTACGAGCTGCCTGAC |
| *β-actin Rev* | AGCACTGTGTTGGCGTACAG |
| Puromycin resistance sequence *Fwd* | GCA ACC TCC CCT TCT ACG AGC |
| Puromycin resistance sequence *Rev* | GCGGGGTAGTCGGCGAAC |
| *NEK 7 Fwd* | CACCTGTTCCTCAGTTCC AAC |
| *NEK 7 Rev* | CTCCATCCAAGAGACAGGCTG |
| *SEC24D Fwd* | TCCGCAGCAGGCAGCCAA |
| *SEC24D Rev* | CCTCTGGTGTTGGTGGCATAAAC |
| *RPL13A Fwd* | GCTTCCAGCACAGGACAGGTAT |
| *RPL13A Rev* | CACCCACTACCCGAGTTCAAG |
| *GBE1 Fwd* | GGAGATCGACCCGTACTTGAA |
| *GBE1 Rev* | ACATCTGTGGACGCCAAATGA |
| *TRIO Fwd* | TTCTTCCGATCCGGGTTTCG |
| *TRIO Rev* | AGACCTCCTCGCTGGGAATA |
| *MAP4K4 Fwd* | TCAAGGGCCAGAATGTGTT |
| *MAP4K4 Rev* | AGTAGGGAGTGCCTATGAACGT |
| *CALD1 Fwd* | CGCCAGAAGATGCCA AAGATG |
| *CALD1 Rev* | TTGGAGACTATTGCTGCTTGATGG |
| *PALM2-AKAP2 Fwd* | CGACCTGCCAATCCTCTGTT |
| *PALM2-AKAP2 Rev* | TCCTTTCTGATG GACCCCCT |
| NEK7 HO *Fwd* | GCACATCACTGGGGACTGAA |
| NEK7 HO *Rev* | GTAGTAGCAATGTGCCGGGT |
| NEK7 CD *Fwd* | ACCTTTCTGTCTTGGAGATGGC |
| NEK7 CD *Rev* | TGCAGCACACCAACATGGC |
| SEC24D HO *Fwd* | GACACCTATGGAAGCTATGCATTT |
| SEC24D HO *Rev* | GCCTGGCCAGTCTTCAGTG |
| SEC24D CD *Fwd* | GTGTACAGCATTGTGCCTGG |
| SEC24D CD *Rev* | AGGACCTGAAGCCCATCTCA |
| GBE1 HO *Fwd* | TGCCCAGACACTGGCAAACA |
| GBE1 HO *Rev* | TGCATTTCACGAGCTTCCTCA |
| GBE1 CD *Fwd* | TTAGGGCCCCTCTTTTGTGG |
| GBE1 CD *Rev* | TCACTGCATGTCTCCGCATC |
| TRIO HO *Fwd* | TGCTATCTTGTGGCCAGGTG |
| TRIO HO *Rev* | CGTCCACAAAACAGATGGCA |
| TRIO CD *Fwd* | AAGAGCTGTCCCTCTGCCAT |
| TRIO CD *Rev* | AGACAGGGCTCCTGATAGCC |
| MAP4K4 HO *Fwd* | CGTGCCTGGCATGTCATCTT |
| MAP4K4 HO *Rev* | GCCTCTACGGCCCATACATAC |
| MAP4K4 CD *Fwd* | GGGACTGTGGTGTTCTTCCC |
| MAP4K4 CD *Rev* | ATGGGCTGTTGGAAACGGTA |
| CALD1 HO *Fwd* | GTAGCACCAGGGCAGTACAA |
| CALD1 HO *Rev* | CAGTTCTCGGTCTGGTGGTT |
| CALD1 CD *Fwd* | CTCAAGGTCTGCTTCAGGGA |
| CALD1 CD *Rev* | CAGCCACCTTGCTTTGTTCT |
| PALM2-AKAP2 HO *Fwd* | GCCAACTGCTGTGATTCTGC |
| PALM2-AKAP2 HO *Rev* | GAGCTCGATGTGAGGAGGTG |
| PALM2-AKAP2 CD *Fwd* | TGTCCACAGATGGTTATTGTCCA |
| PALM2-AKAP2 CD *Rev* | GCTGTTGGGAGGATTCAGTG |
| MDM2 *Fwd* | GGTTGACTCAGCTTTTCCTCTTG |
| MDM2 *Rev* | GGAAAATGCATGGTTTAAATAGCC |
