# Supplementary material for: Aicardi-Goutières syndrome-associated gene SAMHD1 preserves genome integrity by preventing R-loop formation at transcription–replication conflict regions
Source: PLoS Genet. 2021 Apr 15;17(4):e1009523. doi: 10.1371/journal.pgen.1009523 (PMC8078737; doi:10.1371/journal.pgen.1009523)
Supplement: Attachment — Submitted filename: Park_Response to reviewers.docx [file pgen.1009523.s009.docx]

**Point-by-point Responses to the Reviewers’ Comments**

(Park et al., PGENETICS-D-20-01541)

We appreciate the reviewers’ valuable and constructive comments of the reviewers on our manuscript.

**Reviewer #1:**

*The manuscript “Aicardi-Goutières syndrome-associated gene SAMHD1 preserves genome integrity by preventing R-loop formation at transcription-replication conflict regions” by Park et al. reports on R-loop increases in fibroblasts of human AGS patients with mutations of distinct genes. Then, bioinformatic studies provide some evidence that SAMHD1 deficiency may delay replication by increasing R-loops near replication origins located in the body of genes and activation of checkpoint signalling pathways. Finally, bioinformatic analyses of TCGA datasetscorrelate SAMHD1 expression with cancer progression. The data are novel, correctly presented and discussed. However, some critical issues are present in the paper that need to be addressed:*

RE: We are grateful for the time and energy you expended on our behalf. In the following sections, you will find our responses to your points and suggestions. Your comments are in italics. We have carefully addressed the issues raised as follows:

***[1-1]*** *DRIPseq data: it would be interesting to present and discuss the overall distribution of R-loops in the genome of fibroblasts bearing different mutated AGS genes. Is the overall distribution changed with specific gene mutations? In other words: the changes reported in Fig 1 are unique or other changes are present elsewhere?*

RE: Changes in the overall distribution of R-loops in fibroblasts bearing different mutated AGS genes have been presented in a previous report [1], where we adopted the DRIP-seq dataset for AGS patient fibroblasts. Interestingly, AGS-specific R-loops are quite different from the conventional R-loops, which are observed at the transcription start site, transcription termination site, and GC-skew. For example, mutations in RNASEH2A and RNASEH2B genes increased R-loops in the intergenic region and LINE. R-loops are enriched in the gene body region in fibroblasts bearing SAMHD1- and TREX1- mutations (**Figure 3D** of Lim et al., eLife, 2015;4). According to your suggestion, we have added a discussion regarding this issue in our revised manuscript.

**Figure 3D** from Lim et al., eLife, 2015;4 shows the overall distribution of R-loops in fibroblasts bearing different mutated AGS genes.

***[1-2]*** *AGS gene mutations might have an effect on gene transcription and activated ORI may be different among cell types. Thus, some validations of the DRIP-seq results should be provided at single-gene levels. At selected genes, authors should check if their expression levels, ORI activity and R-loop levels are altered (or not), and discuss the results with respect to DRIP-seq findings.*

RE: Thank you for your valuable suggestion. We agree that there is a need for DRIP-seq analysis validation. In Figure 3H of the revised manuscript, we have validated the DRIP-seq result by DRIP-qPCR using two selected genes, NEK7 and SEC24D. We further divided the gene body regions into HO TRC and CD TRC regions, according to the dataset we adopted from Hamperl et al., Cell. 2017;170(4):774-86 e19 [2]. At RPL13A (positive control region), NEK7 HO, and SEC24D HO, R-loop accumulation was enriched in SAMHD1-depleted cells in comparison to that of control cells. We confirmed that the transcription levels of these genes were not altered by SAMHD1 depletion, via RT-qPCR. Additionally, to confirm that the ORI activities of the selected genes were not affected by the depletion of SAMHD1, we determined the relative amount of genomic DNA for selected genome regions by qPCR in SAMHD1-depleted and control U2OS cells (Figs S4G and S4H of the revised manuscript).

***[1-3]*** *Cellular data of Fig 2 are not fully convincing. Panel A reports a difference from 3.5% to 1.7%. Can that be an experimental fluctuation? Is such a difference biological relevant? Moreover, double thymine block is known to be very stressful for cells, inducing DNA damage response (also shown in panel E in fig 2). At least, a better discussion of these points should be included in the text.*

RE: To improve the accuracy and reliability of our experimental results, we have repeated the BrdU pulse-chase experiment and quantified and stated the result of statistical analysis between biological replicates (n=3), as shown in Fig 2B of the revised manuscript. We believe that the approximately 50% reduction in the cell population that newly entered G1 phase is biologically relevant.

Thymidine is a widely used inhibitor of DNA replication. Its inhibition is reversible through washing, and it is relatively less toxic to cells compared to other cell cycle synchronization reagents [3]. As both control and SAMHD1-depleted cells were equally treated with thymidine, we believe that there is a very low possibility of obtaining misled experimental data due to double thymidine blocking. Nevertheless, according to your suggestion, we have included a reference and comment regarding this issue in the revised manuscript.

***[1-4]*** *Panel G, Figure 2: are images mis-labelled ?*

RE: We apologize for any inconvenience due to the error. We have corrected this in the revised manuscript.

***[1-5]*** *Activation of DNA damage response should be demonstrated by checking phosphorylation of H2AX, and both ATR and ATM kinases. This is particular interesting as the authors show that R-loops are increased in the half of ORI where replication/transcription head-on collision may occur.*

RE: Thank you very much for your constructive suggestion. We have included the immunofluorescence assay data for both phospho-ATR and -ATM in SAMHD1-depleted and control cells (Fig 2I–L in the revised manuscript). We observed both phosphor-ATR and –ATM induction in SAMHD1-depleted cells. Even though HO-oriented TRCs promote ATR activation, which responds to ssDNA, while CD-oriented TRCs promote ATM activation, which is primarily activated by DSBs [2], cellular R-loop processing may convert an R-loop to a DSB [4]. Therefore, this result is in agreement with a previously proposed molecular mechanism of R-loop-mediated DNA damage response. We have also discussed these results in our revised manuscript.

***[1-6]*** *Immunofluorescence microscopy images are very poor. Looking the images, I am not convincing that R-loops increase in the nucleus of SAMHD1-deficient cells. I know that immunofluorescence microscopy with S9.6 may be tricky, however, I would suggest not to increase the fluorescence intensity of images to avoid to decrease signal/background ration. In addition, RNaseH1 treatment samples should be shown along with controls in which the same treatment was performed but without RNaseH1 addition in the buffer.*

RE: We have improved and replaced the S9.6 immunofluorescence microscopy image (Fig 3C and 3D in the revised manuscript). First, we changed the experimental conditions to compare the S9.6 signal between SAMHD1-depleted and control cells. Our hypothesis was that TRC-dependent R-loops accumulate in SAMHD1-depleted cells, particularly in the S phase where active transcription takes place. Therefore, we synchronized SAMHD1-depleted and control cells to the S phase and probed with anti-S9.6 for comparison. In addition, we used a microscope with a much higher resolution (inverted microscope Nikon Eclipse Ti2). We also improved the quantification method using custom code written in MATLAB. These changes in the experimental process are described in more detail in the revised manuscript.

For RNaseH treatment, we think that there was a misleading indication about our experimental methods in the original manuscript. Actually, we have treated the samples with the same buffer and in the same final volume, but only with or without RNaseH enzyme. We have changed the term "treated” to “incubated with or without RNaseH” in figure legends and materials & method section in the revised manuscript.

***[1-7]*** *With respect to the previous point, how may an overall R-loop increase (as seen in immunofluorescence images) compare with a rather selective and modest increase at ORIs in transcribed genes?*

RE: As we discussed in the response to ***[1-1]***, the changes in the overall distribution of the R-loop in AGS patient fibroblasts have been previously reported by Lim et al. [1]. DRIP-seq peaks unique to fibroblasts bearing AGS gene mutations may consist of a small portion of total R-loops over the whole genome. Nevertheless, the number of DRIP-seq peaks and the covering length over the genome for AGS patient fibroblasts are much greater than that of healthy control fibroblasts, as previously discussed (**Figure 3A and 3C** of Lim et al., eLife, 2015;4) [1]. Therefore, these gene mutations are certainly “burdens” to cells due to the high load of R-loop in the genome. We have addressed this issue in the Discussion section of our revised manuscript.

**Figure 3A and 3C** from Lim et al., eLife, 2015;4 shows number of DRIP peaks and DRIP peak covering length in fibroblasts bearing different mutated AGS genes.

In addition, according to our new data showing increased transcription–replication collisions in SAMHD1 knockdown cells by proximity ligation assay between RNA polymerase II (RNAPII) and PCNA (Figure 3A-B and 4G-H of revised manuscript), we strongly believe that the level of enrichment in TRC-dependent R-loops upon SAMHD1 depletion was sufficient for detection by such experimental methods (S9.6 immunofluorescence assay).

***[1-8]*** *I suggest to edit the text for English syntax and fluency.*

RE: Thank you for this valuable suggestion. The revised manuscript has been edited using a professional editing service.

**Reviewer #2:**

*In this manuscript, Park, Ryoo and colleagues report the observation that SAMHD1, a factor mutated in the Aicardi Goutières syndrome, prevents the accumulation of R-loops and protect human cells from head-on transcription-replication conflicts and associated genomic instability. This view is largely based on bioinformatic analyses of published datasets showing that SAMHD1 deficiency, but not related AGS mutations, is associated with increased R-loop levels at a subset of genes containing replication origins. In addition, they provide evidence that SAMHD1-depleted U2OS cells have a delayed S phase accumulate spontaneous DNA damage and activate DNA damage response pathways. Finally, they show that colon cancer patients that have a low expression of either SAMHD1 or RNase H2A have a poor survival. On the basis of these data, the authors propose that SAMHD1 contributes to the resolution of R-loops and prevents genomic instability in cancer cells. Although this model is attractive, the data presented in the manuscript are not sufficient to support it and mechanistic insights in how SAMHD1 could execute this important function is missing. Further work is therefore required to clearly establish that SAMHD1 plays a role in R-loop metabolism.*

RE: We really appreciate your thoughtful comments and recommendations. We have carefully addressed the concerns expressed as follows (your comments are in italics), and by doing so, we believe that the manuscript has been substantially strengthened.

***[2-1]*** *In Figure 1, the authors reproduced the analysis performed by the Cimprich lab (Hamperl, Cell 2017) to compare the distribution of R-loops in genes containing a replication origin in the gene body versus genes that do not contain replication origins. To this end, they used DRIP-seq data generated by the Chedin lab (Lim, ELife 2015) to position R-loops in primary fibroblasts from AGS patients and OK-seq data generated by the Hyrien lab to determine fork direction (Petryk, Nat Commun 2016). This analysis revealed a striking difference in the distribution of R-loops at origin-containing genes in SAMHD1-deficient cells, but not in other AGS mutants. Although this result is intriguing, it is not sufficient to conclude that SAMHD1 resolves R-loops at genes experiencing transcription-replication conflicts. Indeed, it is well established that the position of replication origins varies between cell lines. The Cimprich lab mapped R-loops in HeLa cells and analyzed their distribution relative to forks using OK-seq data also generated in HeLa cells. Here, the authors compared R-loops mapped by the Chedin lab in primary fibroblasts relative to forks mapped in HeLa cells, which is like comparing apples and pears. Moreover, SAMHD1-deficient cells have altered dNTP pools and slower replication forks, which may also impact on the replication program and explain the difference with other AGS mutants. In conclusion, although the difference observed between SAMHD1-deficient fibroblasts and other AGS mutants is clear, this analysis needs to be performed in the same cell lines, or at least confirmed for a subset of loci by other methods.*

RE: Thank you very much for your comments. At the beginning of our study, we also had concerns about analyzing datasets obtained from different cell lines. However, a study in 2012 reported that the replication origin distribution was conserved between primary fibroblasts (IMR-90) and HeLa cells (**Supplementary Fig 3a-c** of Besnard et al., Nat Struct Mol Biol. 2012;19(8):837-44.) [5]. In the article, the authors mapped replication origins in multiple cell lines by deep sequencing of short nascent strands and showed that the number and position of peaks are conserved between multiple cell types. The authors said, “Furthermore, origin positions were highly conserved among different cell lines (Fig. 2e), and also when compared with HeLa cells (Supplementary Fig. 3a–c).…”

**Supplementary Fig 3a-c** of Besnard et al., Nat Struct Mol Biol. 2012;19(8):837-44. shows overlapping replication origin distribution between multiple cell lines including primary fibroblasts (IMR-90) and HeLa cells.

SAMHD1 is well known for its dNTPase activity. However, in a previous report by Coquel et al. [6], the authors showed that the role of SAMHD1 in S phase (replication defect) is not limited to the regulation of dNTP pools.

To address your concerns regarding our DRIP-seq analysis in Figure 1, we have confirmed the bioinformatics analysis by DRIP-qPCR (R-loop enrichment), RT-qPCR (transcription levels), and qPCR (replication origin activities) for selected gene bodies and regions (Figs 3H, S4G, and S4H in the revised manuscript). At the selected genes, NEK7 and SEC24D, enriched R-loop accumulation was observed in SAMHD1-depledted cells, in particularly in their HO collision regions (NEK7 HO and SEC24D HO). However, we observed no evidence for any alterations in transcription levels or replication for the selected genes and genomic regions. We hope that our discussion and new data sufficiently support our findings.

***[2-2]*** *The authors claim that they report for the first time a role for SAMHD1 in S-phase progression in unchallenged cells but this statement is incorrect. Other groups have already reported that cell cycle progression is delayed and forks are slower in the absence of SAMHD1 (see for instance Franzolin, PNAS 2013; Kretschmer, Annals of the Rheumatic Diseases 2015; Coquel, Nature 2018). This statement should therefore be toned down. Along the same line, the authors repeatedly state that SAMHD1 is the only AGS-associated gene involved in cancer. RNase H2B has been reported as a colorectal tumor suppressor (Aden et al. https://doi.org/10.1053/j.gastro.2018.09.047). RNase H2A is involved in human gliomagenesis through the regulation of cell proliferation and apoptosis (Dai et al. https://doi.org/10.3892/or.2016.4802).*

RE: According to your comments, we have revisited the manuscript. Our primary intention was to emphasize that we observed a spontaneous DNA damage response in unchallenged SAMHD1-depleted cells for the first time by synchronizing cells to the S phase, rather than observing it in pooled population [6-8]. We have double-checked the manuscript for any misleading information, and we hope this explanation makes the point much clearer.

We have toned down the statement that SAMHD1 is the 'only’ AGS-associated gene that has been reported in cancer by revising to "it is most frequently” or "it is reported in multiple types of cancer.” Additionally, we have added a discussion about the reported relevance of the link between cancer and RNase H2A/B in Figure 5 in the revised manuscript.

***[2-3]*** *The data showing that SAMHD1-depleted cells have increased R-loop levels (Fig. 3) are not really convincing. The reliability of immunofluorescence to quantify R-loops is questioned in the field and the flow cytometry approach would require positive controls to position the gate. The slot blot is not quantified, lacks positive controls and the number of biological replicates is not indicated. DRIP-qPCR experiments on a set of genes should be performed.*

We have improved and replaced the S9.6 immunofluorescence microscopy image (Fig 3C and D). First, we changed the experimental conditions to compare the S9.6 signal between SAMHD1-depleted and control cells. Our hypothesis was that TRC-dependent R-loops accumulate in SAMHD1-depleted cells, particularly in the S phase where active transcription takes place. Therefore, we synchronized SAMHD1-depleted and control cells to the S phase and probed with anti-S9.6 for comparison. In addition, we used a microscope with a much higher resolution (inverted microscope Nikon Eclipse Ti2). We also improved the quantification method using custom code written in MATLAB. These changes in the experimental process are described in more detail in the revised manuscript.

For the flow cytometry approach, we have replaced previous data with Figs 3E, 3F, and S4A–D, according to the comments and suggestions from you and reviewer #3 **[3-5]**. We used siRNAs targeting SAMHD1, and other well-known R-loop regulatory factors such as SETX, THOC1 and MRE11 as positive controls. We performed HB-GFP retention assay with PI co-staining, to observe R-loop enrichment in S phase population of U2OS cells treated with the siRNA. SAMHD1 knockdown cells exhibited increased HB-GFP positive population to the level similar to that of other positive controls, in comparison to that of siControl treated cells (Fig 3E). Additionally, we observed approximately 1.5 fold higher percentage of HB-GFP positive S phase cells in SAMHD1 knockdown cells and in cells treated with siRNA targeting positive controls, in comparison to that of siControl treated cells (n=3) (Fig 3F).

For dot blot analysis, we attempted to include positive controls such as SETX, THOC1, and MRE11 as attached below (**Fig 1 for the reviewer**) using siRNAs.

**Fig 1 for the reviewer: A,** U2OS cells were treated with control (siCon) or indicated siRNAs. At 72 h post knockdown, cells were harvested and subjected to genomic DNA preparation. Half of the genomic DNA was treated with 2 U of RNase H per microgram of genomic DNA. **B,** Quantification analysis for dot blot result shown in **A**. Data represent mean ± SEM, n = 2, P values were calculated using two-tailed Student’s *t*-test.

siSAMHD1 treated U2OS cells showed statistically significant increase in an S9.6 dot blot intensity similar to cells treated with siRNA targeting other positive controls, such as siTHOC1 and siMRE11, in comparison to siCon treated cells. However, we think that the effect of siRNAs are transient and not sufficient for R-loop accumulation to a level of clearer detection by dot blot. Dot blot analysis in siRNA-treated cells could be improved by performing dot blot following S9.6 enrichment of R-loops as previously shown by Cristini et al. [9]. Thus, we have stated the number of biological replicates and quantified the original dot blot data for SAMHD1-depleted cells using shRNA constructs (Fig 3G), which can more stably express and deplete target proteins. We hope that our effort makes this data convincing.

In addition, as you and reviewer #1 suggested in **[1-2]**, we have performed DRIP-qPCR in selected genomic regions and added the new data in the revised manuscript (Fig 3H supplemented with S4G and S4H Figs). We have validated the DRIP-seq result by DRIP-qPCR using two selected genes, NEK7 and SEC24D. We further divided the gene body regions into HO TRC and CD TRC regions. At RPL13A (positive control region), NEK7 HO, and SEC24D HO, R-loop accumulation was enriched in SAMHD1-depleted cells in comparison to that of control cells.

***[2-4]*** *From the data shown in Fig.1, one would predict that the overall amount of R-loops in SAMHD1-deficient cells should not dramatically change if the effect is restricted to genes containing active origins. This issue needs to be clarified textually.*

RE: Thank you very much for your constructive suggestion. DRIP-seq peaks unique to fibroblasts bearing AGS gene mutations may consist of a small portion of total R-loops over the whole genome. Nevertheless, the number of DRIP-seq peaks and the covering length over the genome for AGS patient fibroblasts are much greater than that of healthy control fibroblasts, as previously discussed (**Figure 3A and 3C** of Lim et al., eLife, 2015;4) [1]. We strongly agree with the authors of this previous report who claim that these gene mutations are certainly “burdens” to cells due to the high load of R-loop in the genome.

**Figure 3A and 3C** from Lim et al., eLife, 2015;4 show number of DRIP peaks and DRIP peak covering length in fibroblasts bearing different mutated AGS genes.

In addition, according to our new data showing increased transcription–replication collisions in SAMHD1 knockdown cells by proximity ligation assay between RNA polymerase II (RNAPII) and PCNA (Figure 3A-B and 4G-H of revised manuscript), we strongly believe that the level of enrichment in TRC-dependent R-loops upon SAMHD1 depletion was sufficient for detection by such experimental methods (S9.6 immunofluorescence assay). We have addressed this concern in the Discussion section of our revised manuscript.

***[2-5]*** *In Fig. 4, the authors convincingly show that the overexpression of RNase H1 reduces the formation of spontaneous DSBs in SAMHD1-depleted cells but they would have made their case stronger by showing that these breaks occur during S phase. Moreover, they need to show that the slower S phase and increased gamma-H2AX levels observed in the absence of SAMHD1 (Fig. 2) is also rescued by RNase H1. The authors could also assess fork progression in SAMHD1-depleted cells and test whether fork progression defects can be suppressed by transcription inhibition or RNase H1 overexpression.*

RE: We have now added experimental data for the S phase progression (Fig 4A) and gamma-H2AX levels (Figs 4E and 4F). As expected, RNaseH1 overexpression rescued the slower S phase progression and increased gamma-H2AX level in SAMHD-depleted cells. Thank you for your suggestion.

Regarding your suggestion for fork progression in SAMHD1-depleted cells, it has been presented in a previous study (**Fig 2a and 2c** of Coquel et al., Nature. 2018;557(7703):57-61.) [6].

**Fig 2a and 2c** from Coquel et al., Nature. 2018;557(7703):57-61. SAMHD1 localization at replication forks foci and defects in replication fork progression in SAMHD1-depleted cells.

Therefore, instead of assessing fork progression, we investigated whether SAMHD1-depleted cells experience more transcription–replication collisions by proximity ligation assay (PLA) between RNA polymerase II (RNAPII) and PCNA. We believe that this approach can be a more direct readout to support our hypothesis (Figs 3A and 3B in the revised manuscript). As expected, SAMHD1 depletion increased cellular transcription-replication collision. Additionally, according to your suggestion, we have rescued the increased transcription–replication collision in SAMHD1-depleted cells by RNase H1 overexpression (Figs 4G and 4H in the revised manuscript).

***[2-6]*** *The association of cancer patient survival to SAMHD1 level of expression is interesting, but can it be generalized to other types of cancers? Do tumors expressing low levels of SAMHD1 and RNase H2A constitute different groups? It has been reported that SAMHD1 levels impact on the response to chemotherapy, notably when nucleoside analogues (ara-C) are used (Herold, Nat Med 2017). Is that relevant to colon adenocarcinoma treatment?*

RE: We have not investigated the survival rate for patients with other types of cancers regarding SAMHD1 expression levels. Therefore, at this moment, we cannot generalize the relationship between SAMHD1 expression level and cancer patient survival. However, as SAMHD1 is reported in multiple types of cancers and malignancies [10-15], we believe there could be other cancer types with the association of patient survival to SAMHD1 expression levels.

We have analyzed dataset from 585 colorectal cancer patients to investigate the expression levels of both SAMHD1 and RNase H2A. There are 191 patients with SAMHD1 low expression and 118 patients with RNase H2A low expression. Among those low-expressers, 39 patients overlap as SAMHD1 and RNase H2A low expressers.

As many cancer-relevant SAMHD1 mutants are expression defective [16], we believe that the reported effect of low SAMHD1 expression in response to chemotherapy could be related to our findings. It would be an interesting future investigation to determine the R-loop regulatory function of SAMHD1 in cancer therapy using nucleoside analogues (ara-C).

***[2-7]*** *Fig. S1C: the p-value for DRIP-seq enrichment at CD collision is labelled as P=0.0707, however in the text, the authors indicate P=0.707 (line 158). Which value is correct?*

RE: We apologize for any inconvenience due to the error. We have corrected this in the text of revised manuscript (“P=0.0707").

***[2-8]*** *Fig. 2G: Control cells show a very high gamma-H2AX signal. The labeling is incorrect.*

RE: We apologize for the error. We have corrected this in the revised manuscript.

***[2-9]*** *Fig. 4C: The blot for p-CHK2 should be improved.*

RE: We have improved the image by repeating the experiment using the same sample.

**Reviewer #3:**

*In the manuscript entitled “Aicardi-Goutières syndrome-associated gene SAMHD1 preserves genome integrity by preventing R-loop formation at transcription-replication conflict regions”, the authors show first in a bioinformatic analysis that R-loop levels are increased at particular gene body regions prone to Head-ON transcription-replication conflicts in fibroblasts mutated in the dNTPase and phosphorolytic 3ʹ- 5ʹ -exoribonuclease SAMHD1 compared to control cells (Figure 1). Depletion of SAMHD1 in U-2OS cells leads to replication stress and DNA damage in S-Phase (Figure 2) which correlates with cellular R-loop accumulation (Figure 3). Removal of R-loops can rescue these effects (Figure 4) suggesting a more direct link between R-loop accumulation and the genomic instability phenotypes of SAMHD1 depleted cells. Finally, TCGA database analysis reveals a correlation between SAMHD1 expression level and patient survival rate (Figure 5). Overall, I think the manuscript addresses an interesting question relevant to a broad audience. The data are generally of high quality and convincing. However, I do have a few major concerns that need to be addressed and solidified prior to publication in Plos Genetics.*

RE: Thank you for your constructive comments. We have carefully gone through the comments and suggestions (in italics) and responded to them individually as follows:

***[3-1]*** *The bioinformatic analysis was adapted from a previous study showing that genomic regions prone to HO conflicts have higher R-loop levels than CD (Hamperl et al., Cell 2017). This study used OK-Seq data in HeLa cells to determine replication fork directionality. As I could not find specific information in the material and method section, did the authors use the same data set (derived from HeLa) to determine the locations of origins for the analysis in the AGS mutant cell lines? As usage of replication origins is stochastic and may vary to a significant extent between different cell types and genetic backgrounds, it is unclear to me whether one can confidently conclude that these origins mapped in HeLa cells also exist in the fibroblast cell lines. It would be best if OK-Seq data are available in the exact same cell lines. At the minimum, the authors could analyze additional OK-Seq data sets from other human cell lines and then analyze if and to what extent these origins are conserved between multiple cell lines.*

RE: Thank you very much for your comments. At the beginning of our study, we also had concerns about analyzing datasets obtained from different cell lines. Unfortunately, we could not find any OK-seq data previously analyzed in humans. However, a study in 2012 reported that the replication origin distribution between primary fibroblasts (IMR-90) and HeLa cells was conserved (**Supplementary Fig 3a-c** of Besnard et al., Nat Struct Mol Biol. 2012;19(8):837-44.) [5]. In the article, the authors mapped replication origins in multiple cell lines by deep sequencing of short nascent strands and showed that the number and position of peaks are conserved between multiple cell types. The authors said, “Furthermore, origin positions were highly conserved among different cell lines (Fig. 2e), and also when compared with HeLa cells (Supplementary Fig. 3a–c).…”

**Supplementary Fig 3a-c** of Besnard et al., Nat Struct Mol Biol. 2012;19(8):837-44. shows overlapping replication origin distribution between multiple cell lines including primary fibroblasts (IMR-90) and HeLa cells.

To address your concern regarding our DRIP-seq analysis in Fig 1, we have confirmed the bioinformatics analysis via DRIP-qPCR for selected genes (NEK7 and SEC24D) (Fig 3H). We further divided the selected genes into HO collision and CD collision regions (NEK7 HO/CD and SEC24D HO/CD). In SAMHD1-depleted cells, S9.6 IP signals were enriched at RPL13A, NEK7 HO, and SEC24D HO regions, in comparison to control cells. Additionally, we have checked replication activity and transcription levels in these regions (S4G and S4H Figs in the revised manuscript). We hope that our discussion and the new data included sufficiently support our findings.

***[3-2]*** *Another point regarding this analysis in Figure 1. Have the authors looked into gene expression levels of these 500 genes whether they show significant levels of transcription in the different mutants versus control cells?*

RE: Thank you for your suggestion. We have added RNA-seq analysis data for AGS patient fibroblasts [1] in S1B Fig in our revised manuscript. There were only a few differentially expressed genes between healthy control fibroblasts and other AGS-associated gene mutation-bearing fibroblasts, including SAMHD1-mutated patient fibroblasts (AGS5). We have also added a description regarding this issue in our revised manuscript.

***[3-3]*** *Based on the phenotypes observed in U-2OS cells (Figures 2-4), the authors have clear indications that SAMHD1 knockdown leads to DNA damage-inducing accumulation of RNA:DNA hybrids on a global/cellular level but there is no effect in the overall level of R-loops in SAMHD1 mutant cells compared to control cells (Figure S1A). Is this an effect of knocking down the protein (in U-2OS cells) versus expressing a mutant version of the enzyme in the fibroblast that can induce a dominant negative effect? I think it would be important if the authors could confirm their findings in U-2OS cells by introducing the exact same mutation of SAMHD1 in U-2OS cells in order to make the two different experimental systems/cell lines more comparable. A major selling point of the paper is the relevance of SAMHD1 towards many types of cancer but I believe this point is only proven if the same effects of R-loops at sites of transcription-replication conflicts can be shown with the cancer relevant mutated version of the enzyme.*

RE: Thank you for your considerable discussion of our study. First, the DRIP-seq dataset (GEO accession number: GSE57353) was obtained from two patient replicates [1], one with SAMHD1 deletion mutation (Deletion Ex12-16 hom) and the other with the expression of SAMHD1 alternative splicing variant (c.1609-1G>C hom). The alternative splicing variant mutation is also known as the SAMHD1 Q548X mutant. The Q548X mutant is frequently found in AGS5 patients [17] and has very low expression levels as many SAMHD1 mutants are expression defective [16]. We experimentally confirmed the Q548X mutant expression level in U2OS cells by transfecting and comparing the expression level of WT- with that of Q548-SAMHD1-expressing vector, as attached below (**Fig 2 for the reviewer**).

**Fig 2 for the reviewer: A,** U2OS cells were transfected with WT- or Q548X-SAMHD1-HA-expressing vector. Cells were harvested at 48 h post transfection and subjected to western blotting. **B,** Transfection efficiency for WT- or Q548X-SAMHD1-HA-expressing vector-transfected U2OS cells were checked via qPCR analysis by reading the puromycin resistance sequence of the expressing vector.

Even though we have focused on R-loop accumulations at transcription–replication collision regions for SAMHD1-mutant-bearing patient fibroblasts, the overall distribution of R-loop accumulation in AGS patient fibroblasts altered and varied due to different gene mutations [1]. For instance, mutations in RNASEH2A and RNASEH2B genes increased R-loops in the intergenic region and LINE. R-loops are highly enriched in the gene body region in fibroblasts bearing SAMHD1 and TREX1 mutations. Moreover, AGS-associated gene mutations cause R-loop enrichment in LTRs (**Figure 3D** of Lim et al., eLife, 2015;4). We have addressed this issue in the Discussion section of our revised manuscript.

**Figure 3D** from Lim et al., eLife, 2015;4 shows the overall distribution of R-loops in fibroblasts bearing different mutated AGS genes.

In addition, according to our new data showing increased transcription–replication collisions in SAMHD1 knockdown cells by proximity ligation assay between RNA polymerase II (RNAPII) and PCNA (Figure 3A-B and 4G-H of revised manuscript), we strongly believe that the level of enrichment in TRC-dependent R-loops upon SAMHD1 depletion was sufficient for detection by such experimental methods (S9.6 immunofluorescence assay).

Together, we believe that there was no misinterpretation due to the dominant negative effect of SAMHD1 protein.

We agree with your idea regarding the need to support our findings with an experiment using cancer-relevant SAMHD1 mutants. We have added new data (S5A–D Figs) and a discussion in our revised manuscript. We identified two SAMHD1 mutants (F59C and T232M) that could not rescue the increased cellular transcription–replication conflicts upon SAMHD1-depletion. The effects of R-loops at these conflict sites in SAMHD1-depleted cells seem to be due to SAMHD1 protein depletion, as well as mutations with expression defects (e.g., Q548X, D207Y, and R226H), inability of nucleic acid binding, and inability to interact with the R-loop regulatory cofactor, as discussed in the Discussion section of our revised manuscript.

We hope that we have successfully addressed your concerns. Thank you.

***[3-4]*** *The DNA damage accumulation in SAMHD1-depleted cells leading to Chk1, Chk2 and yH2AX activation shows nicely an S-phase specific effect of SAMHD1 (Figure 2). The alkaline comet assay shows all kinds of DNA damage including ssDNA and DSBs. Could the authors also perform a neutral comet assay to get a better understanding what type of damage is inflicted upon SAMHD1 loss?*

RE: We have added the neutral comet assay results (Figs S3G and S3H in the revised manuscript). SAMHD1-depleted cells showed increased comet tail moments under neutral conditions, indicating that depletion of SAMHD1 also results in double-stranded DNA. This result is in agreement with the previously proposed molecular mechanism of R-loop-mediated DNA damage response, as we have described in our revised manuscript.

***[3-5]*** *Figure 3: Can the authors show that the accumulation of R-loops is specific to S-phase cells (maybe co-labelling with an EdU pulse)? Alternatively, co-stain with PI to investigate the DNA content of cells that have elevated HB-GFP signal (Figure 3D)?*

RE: We have performed an HB-GFP retention assay followed by PI co-staining. SAMHD1-depleted cells showed approximately 1.5-fold enrichment of GFP-positive S phase cell population compared to that shown by control cells. This new data has been included in the revised manuscript as Fig 3F supplemented with S4A–D Figs.

***[3-6]*** *A more conceptual point is that SAMHD1 was recently shown to associate with active replication forks, thereby promoting degradation of nascent DNA at stalled replication forks by stimulating the exonuclease activity of MRE11, thereby mitigating an autoimmune response (Coquel et al., Nature 2018). I guess one model could be that these sites where SAMHD1 acts on are replication forks stalled due to transcription-replication conflicts. Can the authors confirm this by using a more direct readout that shows elevated levels of conflicts in SAMHD1 knockdown conditions (e.g. proximity ligation assays with RNAPII pS2/PCNA or RNAPII pS2/SAMHD1)?*

RE: We truly appreciate your suggestion. We agree that there is a need for a more direct readout to show the elevated level of transcription–replication collisions in SAMHD1-knockdown cells. We have performed a proximity ligation assay with RNA polymerase II and PCNA (Figs 3A and 3B) and rescued the increased transcription–replication collisions in SAMHD1-depleted cells by RNaseH1 overexpression (Figs 4G and 4H). We strongly believe that these data support our findings.

***[3-7]*** *The yH2AX panels in Fig. 2G seem to be flipped between control and SAMHD1 knockdown cells as based on the images, the shLuc cells would have much higher yH2AX signal.*

We apologize for any inconvenience due to the error. We have corrected this in the revised manuscript.

1.	Lim YW, Sanz LA, Xu X, Hartono SR, Chedin F. Genome-wide DNA hypomethylation and RNA:DNA hybrid accumulation in Aicardi-Goutieres syndrome. Elife. 2015;4.

2.	Hamperl S, Bocek MJ, Saldivar JC, Swigut T, Cimprich KA. Transcription-Replication Conflict Orientation Modulates R-Loop Levels and Activates Distinct DNA Damage Responses. Cell. 2017;170(4):774-86 e19.

3.	Amin MA, Varma D. Combining Mitotic Cell Synchronization and High Resolution Confocal Microscopy to Study the Role of Multifunctional Cell Cycle Proteins During Mitosis. J Vis Exp. 2017(130).

4.	Crossley MP, Bocek M, Cimprich KA. R-Loops as Cellular Regulators and Genomic Threats. Mol Cell. 2019;73(3):398-411.

5.	Besnard E, Babled A, Lapasset L, Milhavet O, Parrinello H, Dantec C, et al. Unraveling cell type-specific and reprogrammable human replication origin signatures associated with G-quadruplex consensus motifs. Nat Struct Mol Biol. 2012;19(8):837-44.

6.	Coquel F, Silva MJ, Techer H, Zadorozhny K, Sharma S, Nieminuszczy J, et al. SAMHD1 acts at stalled replication forks to prevent interferon induction. Nature. 2018;557(7703):57-61.

7.	Kretschmer S, Wolf C, Konig N, Staroske W, Guck J, Hausler M, et al. SAMHD1 prevents autoimmunity by maintaining genome stability. Ann Rheum Dis. 2015;74(3):e17.

8.	Franzolin E, Pontarin G, Rampazzo C, Miazzi C, Ferraro P, Palumbo E, et al. The deoxynucleotide triphosphohydrolase SAMHD1 is a major regulator of DNA precursor pools in mammalian cells. Proc Natl Acad Sci U S A. 2013;110(35):14272-7.

9.	Cristini A, Groh M, Kristiansen MS, Gromak N. RNA/DNA Hybrid Interactome Identifies DXH9 as a Molecular Player in Transcriptional Termination and R-Loop-Associated DNA Damage. Cell Rep. 2018;23(6):1891-905.

10.	Crow YJ, Manel N. Aicardi-Goutieres syndrome and the type I interferonopathies. Nat Rev Immunol. 2015;15(7):429-40.

11.	Clifford R, Louis T, Robbe P, Ackroyd S, Burns A, Timbs AT, et al. SAMHD1 is mutated recurrently in chronic lymphocytic leukemia and is involved in response to DNA damage. Blood. 2014;123(7):1021-31.

12.	de Silva S, Wang F, Hake TS, Porcu P, Wong HK, Wu L. Downregulation of SAMHD1 expression correlates with promoter DNA methylation in Sezary syndrome patients. J Invest Dermatol. 2014;134(2):562-5.

13.	Li M, Zhang D, Zhu M, Shen Y, Wei W, Ying S, et al. Roles of SAMHD1 in antiviral defense, autoimmunity and cancer. Rev Med Virol. 2017;27(4).

14.	Rentoft M, Lindell K, Tran P, Chabes AL, Buckland RJ, Watt DL, et al. Heterozygous colon cancer-associated mutations of SAMHD1 have functional significance. Proc Natl Acad Sci U S A. 2016;113(17):4723-8.

15.	Rodero MP, Crow YJ. Type I interferon-mediated monogenic autoinflammation: The type I interferonopathies, a conceptual overview. J Exp Med. 2016;213(12):2527-38.

16.	Kohnken R, Kodigepalli KM, Wu L. Regulation of deoxynucleotide metabolism in cancer: novel mechanisms and therapeutic implications. Mol Cancer. 2015;14:176.

17.	Rice GI, Kasher PR, Forte GM, Mannion NM, Greenwood SM, Szynkiewicz M, et al. Mutations in ADAR1 cause Aicardi-Goutieres syndrome associated with a type I interferon signature. Nat Genet. 2012;44(11):1243-8.
