# Supplementary material for: Aicardi-Goutières syndrome-associated gene SAMHD1 preserves genome integrity by preventing R-loop formation at transcription–replication conflict regions
Source: PLoS Genet. 2021 Apr 15;17(4):e1009523. doi: 10.1371/journal.pgen.1009523 (PMC8078737; doi:10.1371/journal.pgen.1009523)
Supplement: Attachment — Submitted filename: Park_revised_Response to reviewers.docx [file pgen.1009523.s010.docx]

**Point-by-point Responses to the Reviewers’ Comments**

(Park et al., PGENETICS-D-20-01541R1)

We really appreciate the positive comments from reviewers. Thank you.

**Reviewer #2:**

*Reviewer #2: The authors have made a great effort revising this manuscript, with the addition of numerous new experiments. Overall, these additional data have greatly improved the manuscript, which is now potentially suitable for publication. I still have two concerns and one minor request.*

RE: We really appreciate your positive and constructive comments. We have made best effort to address your concerns as follows.

***[2-1]*** *My first concern is that although the authors have provided new evidence that R-loops levels are higher in SAMHD1-depleted cells, it is a bit disappointed that they analyzed only two genes (NEK7 and SEC24D) by DRIP-qPCR.*

RE: We agree with your suggestion that analyzing more number of genes by DRIP-qPCR can provide evidences supporting our bioinformatics analysis and findings. However, it is not possible to access ~700 genes for their DRIP signals by DRIP-qPCR, we have added 5 more genes. In the other words, we analyzed five more pairs of HO-CD genomic regions. The genes are GBE1, TRIO, MAP4K4, CALD1 and PALM2-AKAP2, which are also actively transcribed genes like NEK7 and SEC24D. As we have described in our revised manuscript (Line 378-386 of revised manuscript_Changes Highlighted), R-loops on HO regions of all those five genes were enriched in SAMHD1-depleted cells than in control cells by more than 1.5-fold. There were genes those showed higher DRIP signals on their CD regions (GBE1, TRIO and CALD1), but the enrichment was marginal and the adjusted P values were much larger than that of their HO regions. Importantly, DRIP signals of those genes on HO regions were much more sensitive to *in vitro* RNaseH1 treatment (S4I fig). Lastly, but importantly, the transcription levels of the five genes were not affected by SAMHD1 protein depletion (S4H fig).

We really hope that our additional data have successfully addressed your concern. Thank you.

***[2-2]*** *The second (major) concern relates to the experiment shown in Fig. S4H to support the view that the same replication origins are used in the absence of SAMHD1. The fact that DNA copy number does not change at loci of interest does not mean that the same origins are used. Changes in DNA copy number in a population of cells indicates at best the time of replication (early or late), not origin usage. Moreover, the replication of the reference locus used to normalize DNA copy numbers could also change in SAMHD1-deficient cells, which would affect the interpretation of the data. In short, this experiment is inconclusive and should be removed. Removal of these data would also weaken the argument that origin usage does not change in the absence of SAMHD1 and this limitation needs to be discussed in the manuscript.*

RE: Thank you very much for your constructive comment. According to your suggestion, we have removed the Fig. S4H. Instead, we have added a discussion in our revised manuscript as “We assumed that the replication origin usages are not altered by SAMHD1 protein depletion, as it is previously observed in AGS fibroblasts and HEK293T cell line [1, 2].” (Line 369-371 of revised manuscript_Changes Highlighted).

***[2-3]*** *Finally, the novel PLA experiments are very nice, but the intensity of PLA foci in Figs 4G and 3A is too low and should be increased.*

RE: We have increased the intensity of PLA foci in Figs 3A and 4G according to your suggestion. We believe it is now much clearer. Thank you.

**Reviewer #3:**

*The authors have addressed all my previous comments/concerns and have also added significant new experimental data including DRIP-qPCR validation of the bioinformatic analysis, S9.6 IF and FACS quantifications with appropriate positive and negative controls as well as PLA data between RNAPII-PCNA as requested by the reviewers. Together, the results substantiate the author’s conclusions that R-loops are highly enriched at transcription-replication conflict regions of the genome in fibroblast of patients bearing SAMHD1 mutation. Therefore, I’m positive and support timely publication of the manuscript in Plos Genetics.*

RE: Thank you very much for your positive reception of the revised manuscript.

1.	Coquel F, Silva MJ, Techer H, Zadorozhny K, Sharma S, Nieminuszczy J, et al. SAMHD1 acts at stalled replication forks to prevent interferon induction. Nature. 2018;557(7703):57-61.

2.	Franzolin E, Coletta S, Ferraro P, Pontarin G, D'Aronco G, Stevanoni M, et al. SAMHD1-deficient fibroblasts from Aicardi-Goutieres Syndrome patients can escape senescence and accumulate mutations. FASEB J. 2020;34(1):631-47.
